# Rhabdomyosarcoma of the Maxilla

**DOI:** 10.5334/jbsr.3797

**Published:** 2024-12-09

**Authors:** Karel Mercken, Maarten Steyvers, Robert Hermans

**Affiliations:** 1 Department of Radiology, University Hospitals Leuven, Leuven, Belgium

**Keywords:** rhabdomyosarcoma, soft tissue sarcoma, maxilla, head and neck, parameningeal, pediatric

## Abstract

Rhabdomyosarcoma is the most common soft tissue sarcoma in children but is less frequent in adults, with the head and neck region as primary site. Magnetic resonance imaging (MRI) is the preferred diagnostic imaging tool, though its imaging characteristics are relatively non‑specific and overlap with other soft tissue sarcomas. The prognosis of rhabdomyosarcoma depends on the primary tumour site and size, with parameningeal head and neck localisations having a less favourable prognosis due to the higher risk of spread. Therefore, further imaging including brain and spinal MRI is recommended.

*Teaching point:* The prognosis of rhabdomyosarcoma depends on the primary tumour site and size, with parameningeal head and neck localisations having a less favourable prognosis due to the higher risk of spread.

## Introduction

Rhabdomyosarcoma (RMS), a striated skeletal muscle malignancy, accounts for 50% of the soft tissue sarcomas in children (5–7% of the pediatric malignancies), while accounting for less than 10% of the adult soft tissue sarcomas [1–2]. The primary anatomical locations include (in descending order) the head and neck region (orbit; parameningeal regions such as the nasal cavity, nasopharynx, paranasal sinuses, temporal bone and pterygopalatine fossa; and non‑parameningeal regions such as the oral cavity, oropharynx, larynx, masseter and so on), genitourinary tract, extremities, thoracic cavity and retroperitoneum [1].

The current World Health Organization (WHO) classification of RMS (2020) includes four main subtypes based on pathological and molecular characteristics: embryonal (70–80% of cases in which 33% between 0 and 5 years and 50% in the head and neck region), alveolar (20% of cases, more frequent in adolescents and young adults; mainly extremities, head and neck region and trunk), spindle cell/sclerosing (including newer variants) and pleiomorphic. Rare subtypes include epithelioid RMS, ectomesenchymoma and RMS in an inflammatory rhabdomyoblastic tumour [3]. Genomic analyses have revealed distinct subcategories [4].

## Case Report

### Case 1

**Figure 1:**
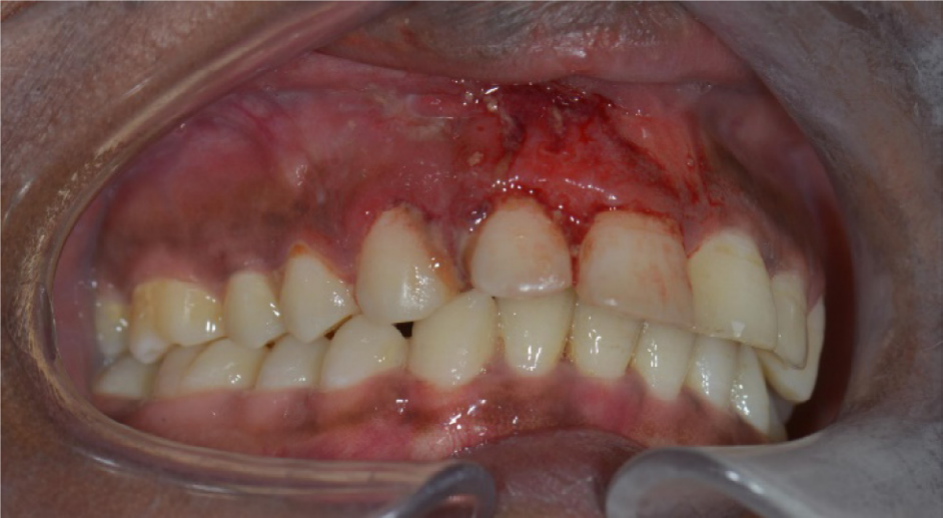
Soft tissue swelling with gingival hyperemia and bleeding in the right maxilla around element 11 and 12.

**Figure 2:**
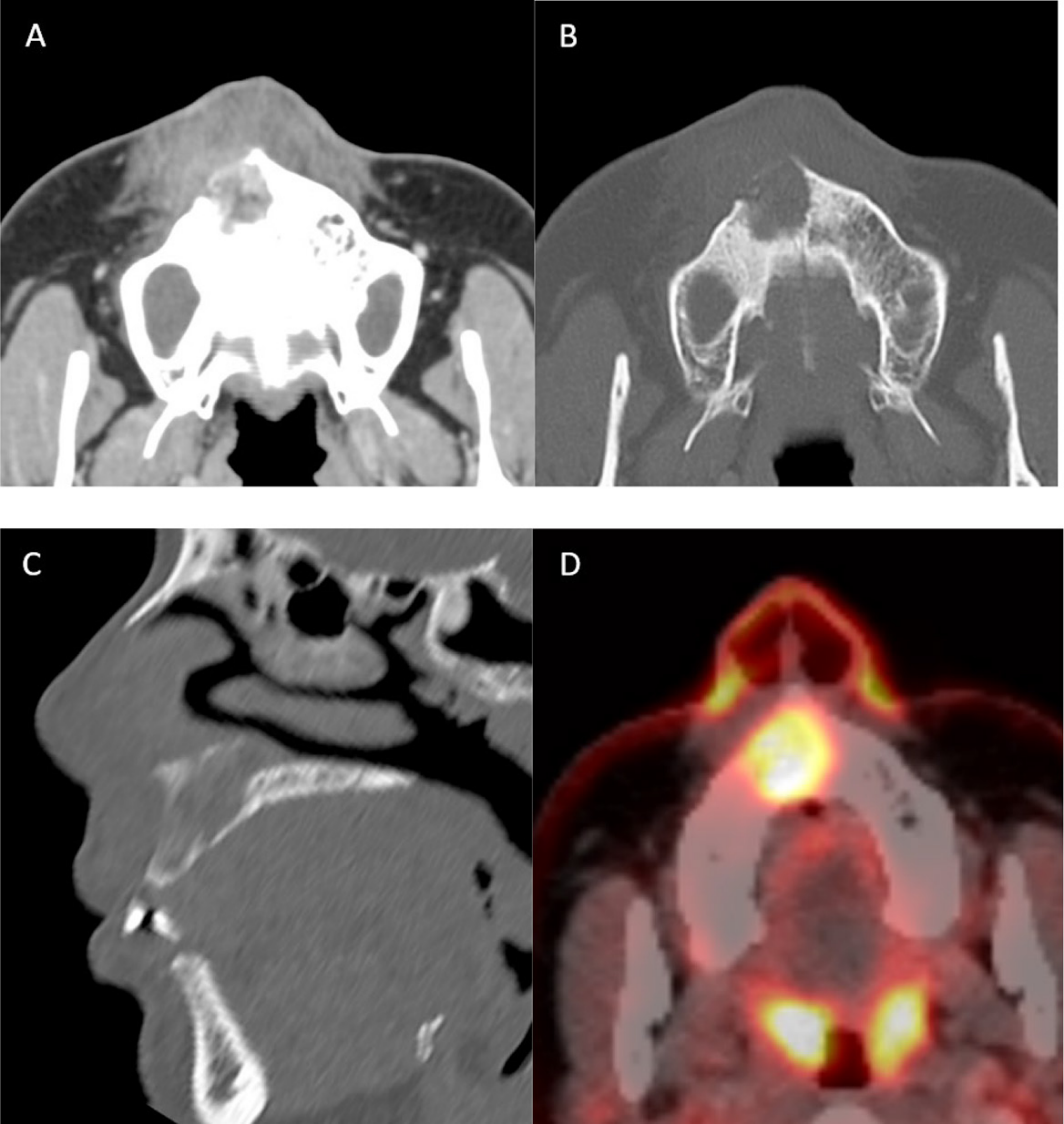
Axial and sagittal CT images (**A–C**) and axial PET–CT image (**D**) showing an osteolytic lesion with heterogeneous contrast enhancement in the right maxilla, with palatal cortical erosion and with high FDG uptake.

A 25‑year‑old woman presented with a mucosal lesion in the right maxilla with intermittent local pain as only complaint (Figure 1). Computed tomography (CT) revealed an osteolytic lesion in the right maxilla, centered cranially to element 11 and 12, associated with buccal and palatal cortical breakthrough (Figure 2A–C). The lesion demonstrated strong fluorodeoxyglucose (FDG) uptake on positron emission tomography (PET)–CT (Figure 2D). Biopsy indicated a spindle cell‑to‑epithelioid‑like tumour. RNA sequencing confirmed the presence of a FUS‑TFCP2 fusion, consistent with RMS with TFCP2 gene rearrangement. A hemimaxillectomy was performed to attempt complete lesion resection after initial maxillary compartment resection and systemic therapy. The long‑term outcome is not yet known.

### Case 2

**Figure 3:**
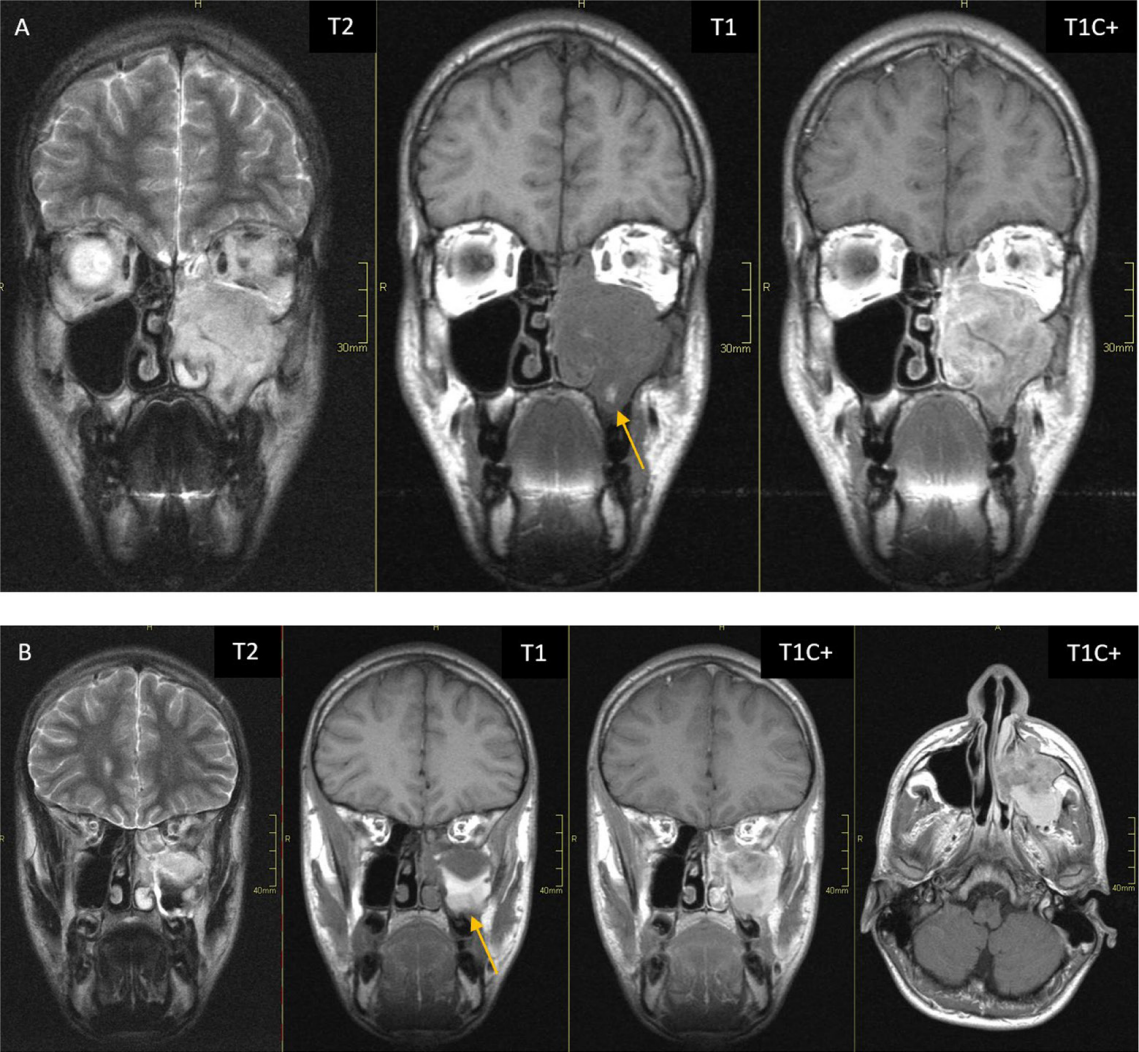
(**A–B**) MRI sequences (T2, T1C(−), T1C(+)) showing a lesion in the left maxillary sinus extending to the nasoethmoidal region and retromaxillary tissues. The tumour appears T2 hyperintense, T1 isointense (with focal T1 hyperintensities due to recent haemorrhage; arrows) and with heterogeneous contrast enhancement.

A 17‑year‑old male presented with a progressively enlarging swelling beneath the left eyelid, accompanied by epiphora, paranasal numbness and transient nasal obstruction. Magnetic resonance imaging (MRI) revealed an expansile mass in the left maxillary sinus and nasoethmoidal region extending into the orbit, the alveolar process of the maxilla and the pterygopalatine fossa. The lesion showed largely homogeneous isointensity on T1‑weighted MRI, heterogenous moderate hyperintensity on T2‑weighted sequences and marked enhancement after gadolinium administration (Figure 3A–B). A spontaneous T1‑hyperintense area within the lesion is consistent with focal haemorrhage. Some T2‑hyperintense inflammatory alterations are seen in the left maxillary sinus (Figure 3B). The immunohistochemical profile was consistent with RMS. Fluorescence in situ hybridisation (FISH) analysis showed no fusion of the PAX3 or PAX7 genes with the FOXO1 gene, excluding the alveolar subtype. RNA sequencing was not performed as the case pre‑dated the implementation of this technique. Given the rapid clinical tumour progression and inoperability, chemoradiotherapy was initiated, leading to fast volume reduction and regression of the lesion. The patient remained relapse‑free.

## Discussion

The MRI characteristics of RMS are similar among its most prevalent subtypes but are relatively nonspecific in soft tissue sarcomas. On T1‑weighted MRI, the lesion appears isointense to hyperintense relative to muscle. T2‑weighted imaging reveals a heterogeneously hyperintense signal, attributable to necrosis, haemorrhage or a myxoid component. Contrast‑enhanced T1‑weighted imaging demonstrates heterogeneous enhancement due to necrotic areas in the lesion. Viable tumour tissue typically shows diffusion restriction. Dynamic contrast‑enhanced imaging often displays early enhancement in the solid tumour areas. Bony destruction is often present [2]. As distant metastases may be present, most commonly in the lungs, a chest CT or FDG–PET study is indicated for appropriate staging [1].

The differential diagnosis for RMS depends on the tumour type, location and size and the patient’s age. Depending on the location in the head and neck region, tumoural lesions such as lymphoma, other types of sarcomata and nasopharyngeal carcinoma should be included in the differential diagnosis. In the retroperitoneum, germ cell tumours are included in this list. In cases where the tumour is found in the extremities or trunk, other soft‑tissue sarcomas such as synovial sarcoma and extraosseous Ewing sarcoma must be taken into account [2].

The prognosis of RMS depends on primary site, tumour size, histological subtype and stage at diagnosis. Parameningeal tumours are associated with poorer outcomes due to the risk of subarachnoid dissemination [1]. The prognosis of embryonal RMS and infantile spindle cell RMS are favourable, while alveolar and pleiomorphic RMS have a poor prognosis [5]. Metastatic RMS has a poorer prognosis with relapse rates up to 70%, unlike 25–30% in nonmetastatic disease [6].

## Conclusions

Rhabdomyosarcoma is the most common soft tissue sarcoma in children but is less frequent in adults, with the head and neck region as primary site. MRI is the preferred diagnostic imaging tool, though its features are relatively non‑specific and overlap with other soft tissue sarcomas. Radiologists should consider rhabdomyosarcoma when identifying a mass in the maxillary region, particularly in youngsters. For tumours in parameningeal locations, further imaging, including brain and spinal MRI, is recommended.
